# Mr. Capper, on the External Use of Laudanum in Hiccup

**Published:** 1801-07

**Authors:** E. Pitts Gapper

**Affiliations:** Ewel, Surry


### *To the Editors of the Medical and Phyſical Journal.*

GENTLEMEN,

I Tranſmit you the incloſed caſe for your valuable Journal, if you ſhould deem it worthy of inſertion. I am, &c.

*Ewel, Surry, June* 9, 1801. E. PITTS GAPPER.

---

ON the 3d of April I was deſired to viſit a young man, of a ſcrophulous habit, who was ill of an inflammatory affection of his lungs, which was accompanied with the uſual ſymptoms of cough, fever, &c. But as the diſeaſe itſelf did not differ in its appearance from what is generally obſerved, until the ſymptom which is the ſubject of this paper came on, I do not think it neceſſary to detail its progreſs, only premiſing that he was bled twice, that the blood was inflamed, but not highly ſo; that bliſters were repeatedly applied to the thorax, and that antimonial, ſaline, and aperient medicines were adminiſtered, as circumſtances required, with a ſtrict attention to an antiphlogiſtic regimen, though without any apparent advantage being gained by it.

On the 19th he was ſeized with a hiccup, the moſt violent, diſtreſſing, and inceſſant I ever witneſſed; it was loud enough to be heard in the ſtreet at the diſtance of ſeveral yards from the houſe, and continued without even ceaſing a ſingle minute for near *eighty hours*, before any relief was obtained. The pulſe, during this period, was ſmall, and fluctuated from 120 to 130 in a minute. I was not remiſs in employing whatever remedies I judged moſt likely to be of ſervice. Muſk, aſſafœtida, amber, æther, volatiles, and opium were given, variouſly combined, at ſhort intervals, and in conſiderable doſes. A large bliſter was applied to the region of the ſtomach, and fomentations to the feet. At the end of the above-mentioned period not the leaſt abatement could be perceived, the hiccup continuing as loud, as violent, and as inceſſant as ever. I began now to deſpair of my patient; when, as every thing elſe had failed, it occurred to me to employ opium by friction, in the manner I had ſeen it recommended in your uſeful Journal, in caſes of delirium attendant on typhus fevers. I therefore ordered, on the evening of the 21ſt, two drachms of tinct. opii, mixed by means of a little yolk of an egg, with the ſame quantity of oil of almonds, to be rubbed into the thighs, and to be repeated every eight hours. Care was taken by my aſſiſtant that it was properly done, and that the friction was continued until the whole was abforbed. When I faw him the next morning the liniment had been twice ufed, and I was much pleafed to find with fome effect. He was much more compofed than on the preceding night, and though the hiccup was ftill as frequent as ever, yet it was lefs violent and loud. The pulfe was alfo reduced to 112. Encouraged by thefe flattering circumftances, I ordered a continuance of the liniment. On the morning of the 22d he was in much the fame ftate as on the preceding day; but, in the evening, I was happy to find that intermiffions, though fhort ones, had taken place. On the next day, the 23d, the intermiffions were increafed in frequency, and were extended to the length of half an hour or more; and the pulfe was reduced to 90. The liniment was continued as before. From this time the hiccup became lefs frequent and violent, and the intermiffions longer, until the 25th, when it wholly left him. The remainder of the cafe, as not being connected with the fubject, it is unneceffary to relate; it is fufficient to fay that my patient ultimately recovered.

During the ufe of the liniment he took no other medicine than the fimple faline draught, and fo far from the bowels becoming conftipated under its ufe, it was not even neceffary to give him any aperient medicine during its exhibition. It occafioned a confiderable degree of drowfinefs, but not in any degree to excite the leaft alarm. The quantity of tinct. opii ufed was two ounces in the fpace of little more than three days; a quantity which could not have been exhibited in any other way without the moft imminent hazard and danger. Such a wonderful effect as is here related naturally leads the mind to fpeculate on the modus operandi, and to difcover why a certain quantity of opium may be conveyed into the fyftem through the abforbents, not only without detriment, but with great and decided advantage, which if admitted into the ftomach would probably occafion death. I fhall not enter into fuch a difquifition, as many and repeated experiments, and much reflection, are neceffary to give a fatisfactory explanation. I fhall only be happy if, in fimilar circumftances, it may prove as efficacious in other hands as it has done in mine.

